# Supplementary material for: Expression of novel long noncoding RNAs defines virus-specific effector and memory CD8+ T cells
Source: Nat Commun. 2019 Jan 14;10:196. doi: 10.1038/s41467-018-07956-7 (PMC6331603; doi:10.1038/s41467-018-07956-7)
Supplement: Supplementary file 1 — Supplementary Information [file 41467_2018_7956_MOESM1_ESM.pdf]

Supplementary Information for

Hudson et al., Expression of novel long noncoding RNAs defines virus-specific effector and memory $CD8^+$ T cells

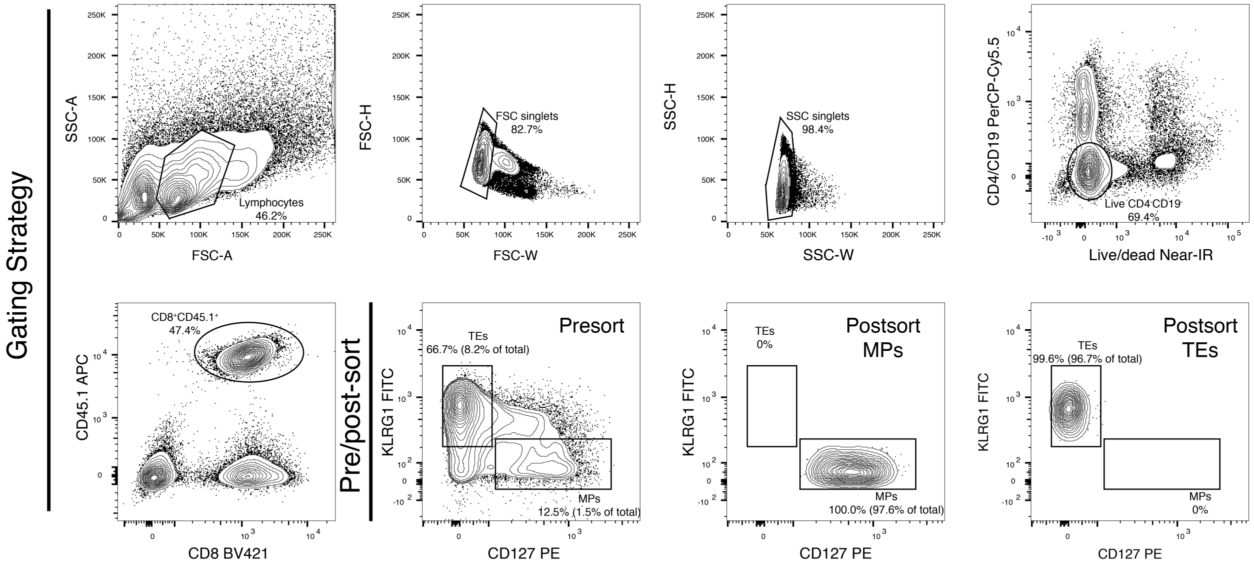

Supplementary Figure 1: Gating strategy for isolating day 8 memory precursor and terminal effector mouse CD8+ T cells. RNA from these cells was used in main text Figures 1-3.

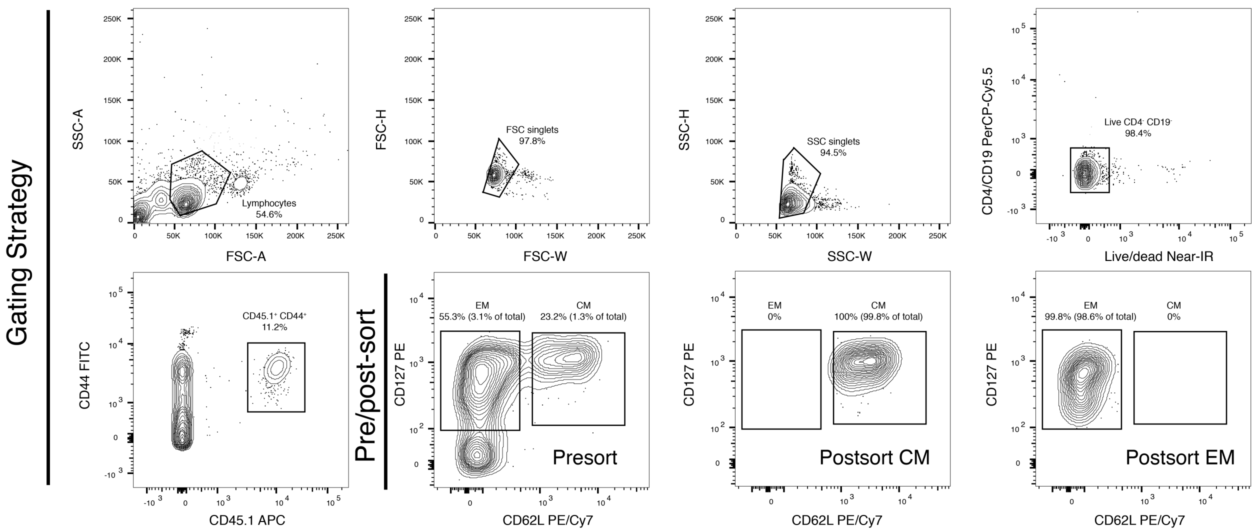

Supplementary Figure 2: Gating strategy for isolating day 48 memory CD8+ T cells. CM, central memory; EM, effector memory. RNA from these cells was used in main text Figures 1-3.

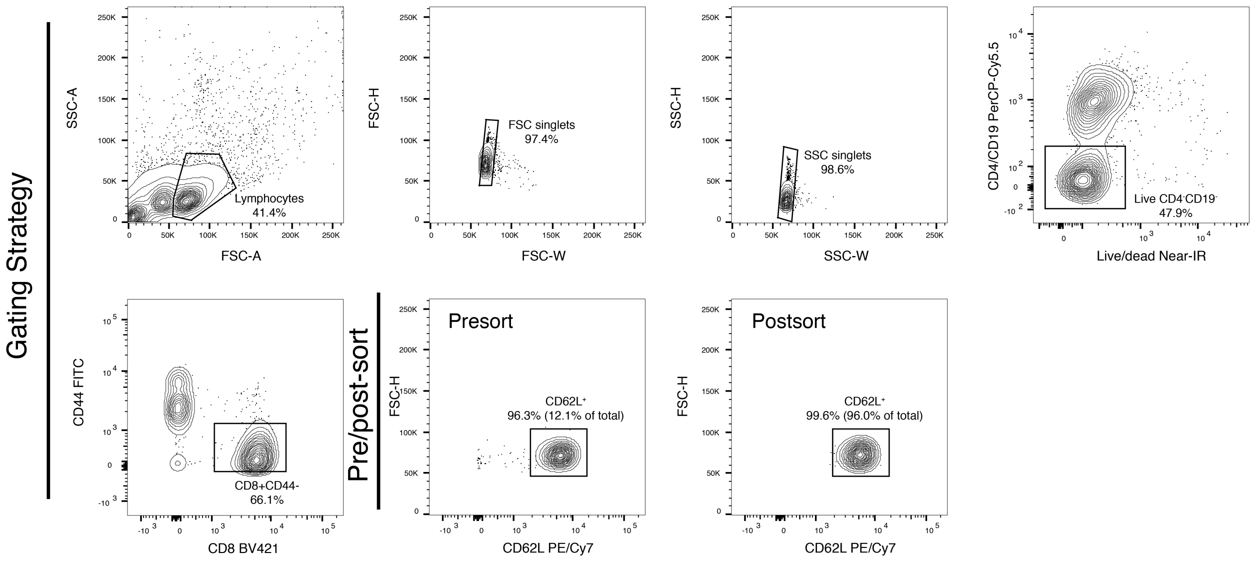

Supplementary Figure 3: Gating strategy for isolating naive CD8+ T cells. RNA from these cells was used in main text Figures 1-3.

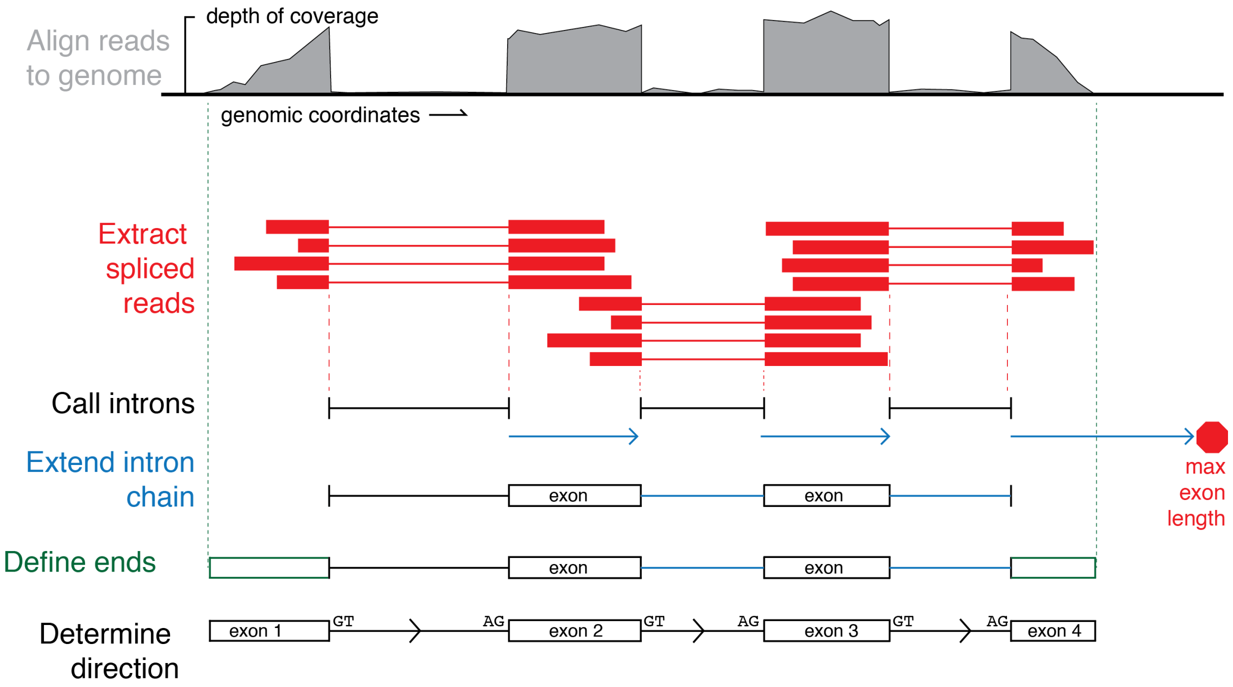

Supplementary Figure 4: Schematic of spliced transcript discovery by intron chain extension (ICE). After alignment of reads to the genome (gray), spliced reads are extracted. From these, introns (splice junctions) are called if a sufficient number of intron-spanning reads are present between two genomic coordinates. From every splice junction, a novel transcript is formed by searching bidirectionally for nearby splice sites within a given maximum exon length; no additional introns are added once this length is reached in each direction. The ends of the final exons are then determined by overall depth of coverage of spliced and unspliced reads. Direction of transcription is determined by searching for consensus splice donor and acceptor sites; transcripts with conflicting direction information are discarded. Transcripts whose intron chains are duplicated or are subsets of another transcript are discarded.

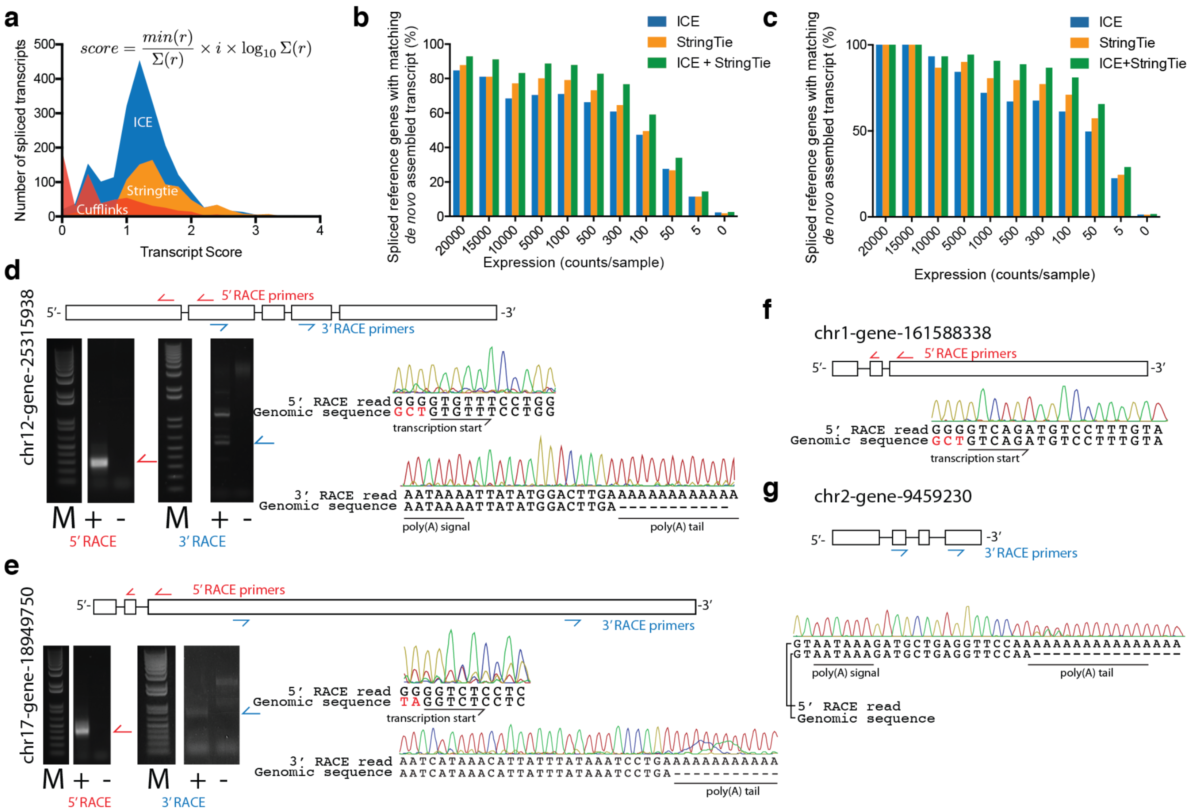

Supplementary Figure 5: Validation of CD8+ T cell *de novo* transcriptome assembly of spliced transcripts. a. Spliced *de novo* transcripts originating from Cufflinks, StringTie, and ICE were scored according to the number of spliced reads supporting each intron. A score of zero indicates at least one called intron with zero supporting RNA-seq reads. **b.** To validate *de novo* transcriptome assemblies from ICE and StringTie, their spliced transcriptomes were compared with the mouse *Ensembl* reference spliced transcriptome. Any *Ensembl* transcript with an intron chain completely matching an ICE, StringTie, or combination transcript was considered matched to the reference genome. **c.** Identical to **b**, but with human *de novo* and reference transcriptomes. **d.** 5’ and 3’ RACE on the novel transcript chr12-gene-25315938, which is highly expressed in effector T cells. **e.** 5’ and 3’ RACE on the novel transcript chr17-gene-18949750, which is highly expressed in naïve CD8+ T cells. **f.** 5’ RACE on the transcript chr1-gene-161588338, which is highly expressed in memory precursor and memory CD8+ T cells. **g.** 3’ RACE on the transcript chr2-gene-9459230, which is highly expressed in memory precursor and memory CD8+ T cells. In panels **e** and **f**, agarose gels of RACE products are shown: M indicates a molecular weight marker, (+) indicates the use of gene-specific and anchor primers, and (-) indicates the use of an anchor primer only as a negative control.

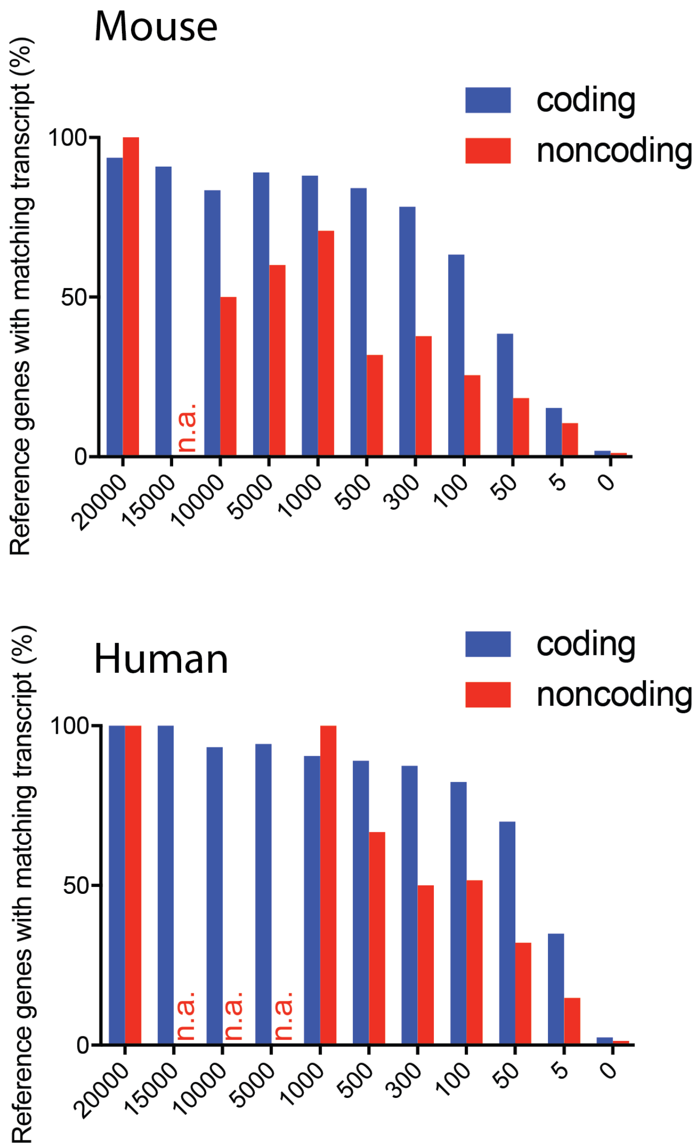

Supplementary Figure 6: ICE and StringTie recapitulation of protein-coding and noncoding reference transcripts. As in Supplementary Figure 5b-c, spliced transcriptomes were compared with the *Ensembl* reference genome annotation. Bars show the percentage of coding or noncoding reference transcripts with at least one isoform matched by our transcriptome. n.a. indicates not applicable, the presence of no annotated transcripts of the given type and expression level.

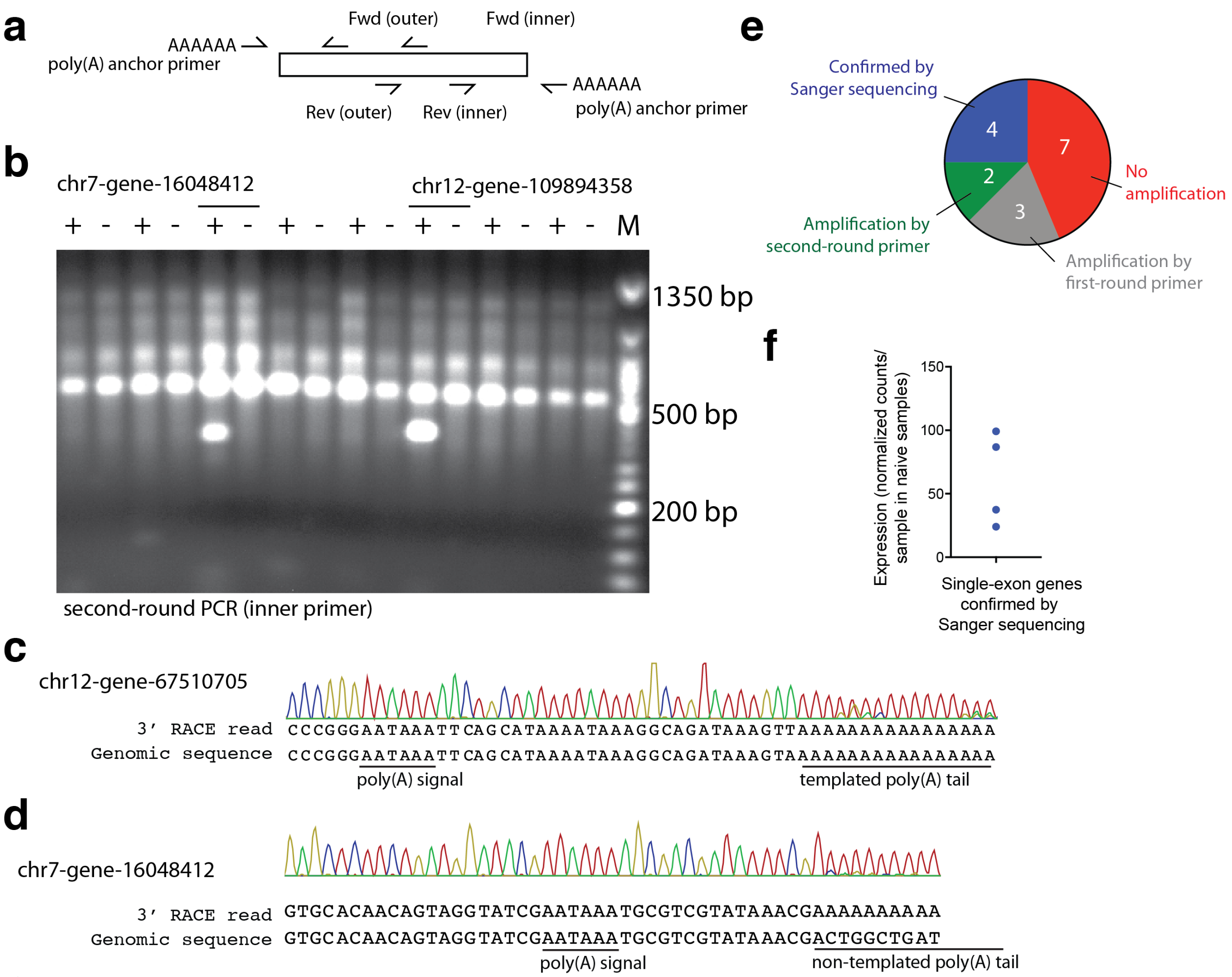

Supplementary Figure 7: Validation of CD8+ T cell de novo transcriptome assembly of single-exon transcripts. a. Using automated methods, we designed two nested primers for each possible direction of transcription for 16 randomly-selected novel single-exon transcripts that were predicted to be 500-1500 nucleotides in length. We performed PCR sequentially with each primer as well as a poly(dT)-based primer on cDNA generated from RNA isolated from naive C57BL/6J mice (separate from the mice used elsewhere in the study), and analyzed results by agarose gel electrophoresis (panel **b**), and when possible, sent results for Sanger sequencing (panel **c, d**). Two confirmed genes are shown as examples, including one pseudogene with a template poly(A) tail (top), and one with a canonical poly(A) signal and non-templated poly(A) tail. **e**. Summary of RACE results of the 16 randomly selected single-exon transcripts. **f.** Expression levels (normalized counts/sample) of the four single-exon genes confirmed by Sanger sequencing.

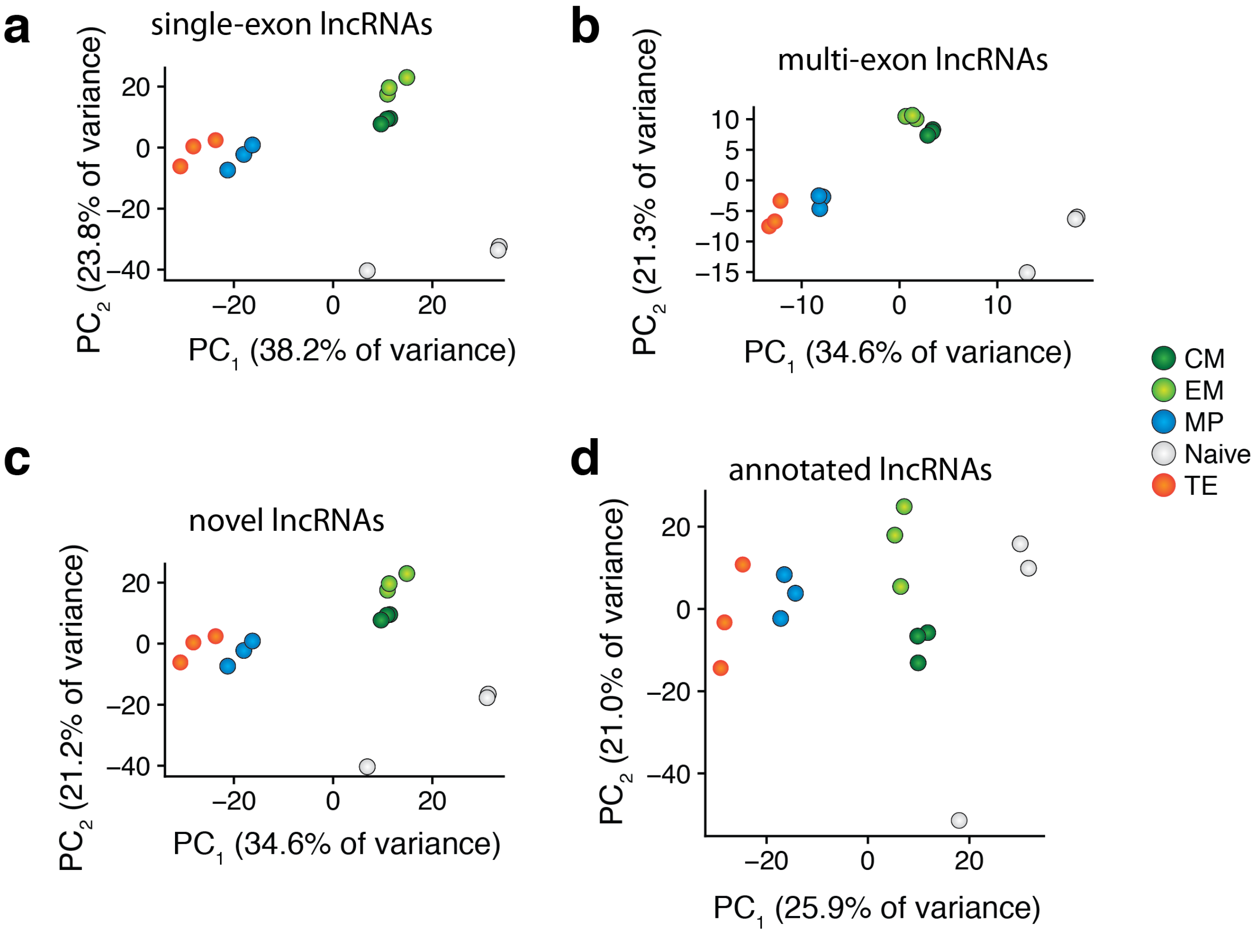

Supplementary Figure 8: Principal component analysis (PCA) using distinct types of lncRNA genes shows similar clustering of CD8+ T cell types. a-b. PCA using expression single- or multi-exon lncRNA genes. Both annotated and novel genes are used. **c-d.** PCA using novel or annotated lncRNAs only. See also Figure 3. All PCA analyses are shown for mouse genes.

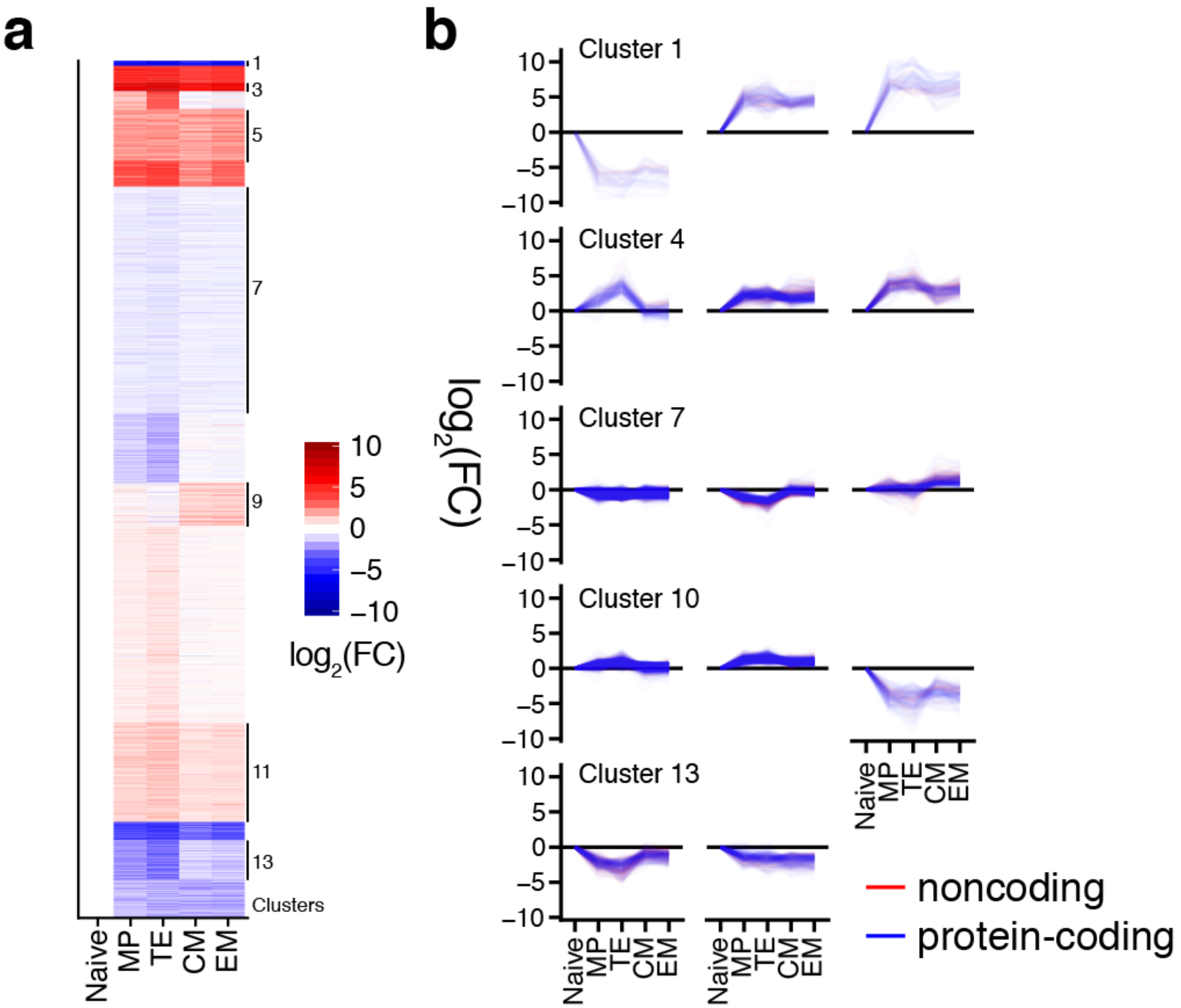

Supplementary Figure 9: Unsupervised clustering of statistically significant differentially expressed genes in mouse CD8+ T cell differentiation. a. Heatmap of all differentially expressed genes in CD8+ T cell differentiation. At right, cluster numbers are shown. **b.** Line graph showing expression of individual genes from each cluster. Noncoding genes are shown in red, protein-coding genes in blue.

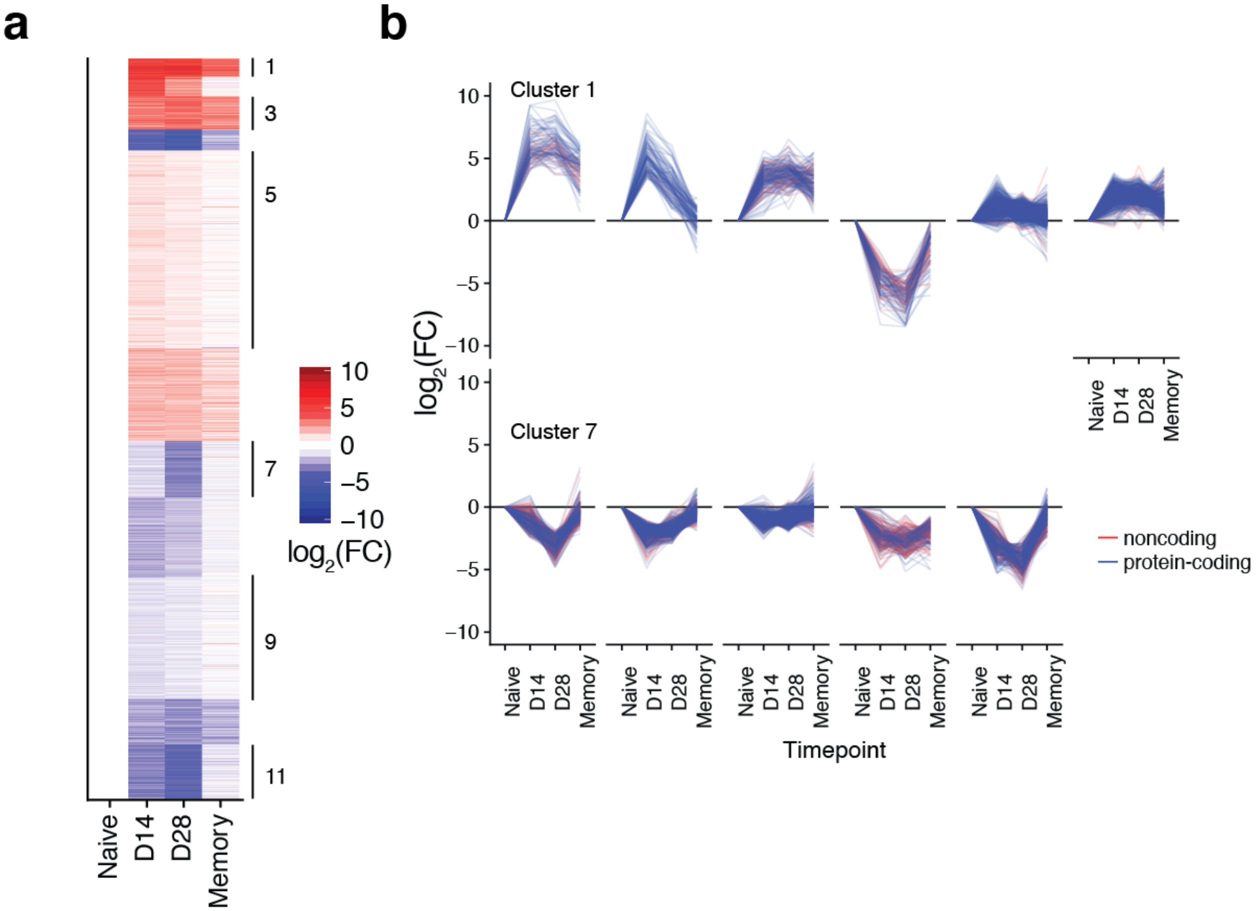

Supplementary Figure 10: Unsupervised clustering of statistically significant differentially expressed genes in human CD8+ T cell differentiation. a. Heatmap of all differentially expressed genes in CD8+ T cell differentiation. At left, cluster number is shown. **b.** Line graph showing expression of individual genes from each cluster. Noncoding genes are shown in red, protein-coding genes in blue.

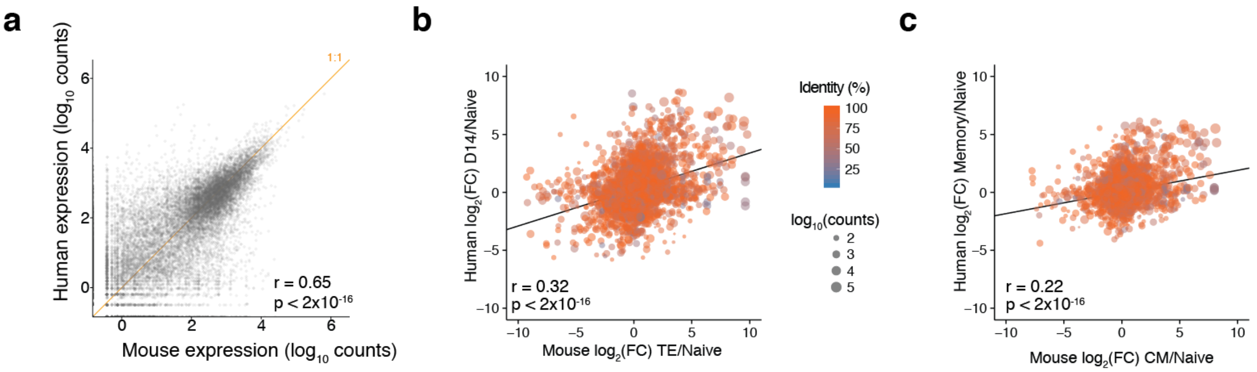

Supplementary Figure 11: Despite expressing a highly similar repertoire of genes, mouse and human $CD8^+$ T cells show only moderate correlations of expression in homologous genes after activation. a. Known mouse-human homolog pairs were retrieved from *Ensembl*. For each pair, mouse and human expression in naïve $CD8^+$ T cells are shown. Read counts have been corrected for sequencing library size. **b.** For each of these pairs, $\log_2$ fold change between peak effector and naïve $CD8^+$ T cells are shown. Percent identity (from *Ensembl*) is shown by color, and average expression across all mouse samples is shown by size. **c.** Identical to panel **b**, with memory cells shown. Pearson's *r* is shown, with p-value calculated using a *t* distribution.
