# Supplementary material for: Expression of novel long noncoding RNAs defines virus-specific effector and memory CD8+ T cells
Source: Nat Commun. 2019 Jan 14;10:196. doi: 10.1038/s41467-018-07956-7 (PMC6331603; doi:10.1038/s41467-018-07956-7)
Supplement: Supplementary file 3 — Description of Additional Supplementary Files [file 41467_2018_7956_MOESM3_ESM.pdf]

Description of Additional Supplementary Files

Supplementary Data 1: Expression levels, DESeq2 results, and clustering results for genes expressed in mouse CD8+ T cells.

Supplementary Data 2: Expression levels, DESeq2 results, and clustering results for genes expressed in human CD8+ T cells.

Supplementary Data 3: GTF of novel mouse genes

Supplementary Data 4: GTF of novel human genes
